# Maternal Dietary Nitrate Supplementation Lowers Incidence of Stillbirth in Hyper Prolific Sows under Commercial Circumstances

**DOI:** 10.3390/ani11123364

**Published:** 2021-11-24

**Authors:** Moniek van den Bosch, Bram Bronsvoort, Bas Kemp, Henry van den Brand

**Affiliations:** 1 Adaptation Physiology Group, Wageningen University and Research, P.O. Box 338, 6700 AH Wageningen, The Netherlands; brambronsvoort@gmail.com (B.B.); bas.kemp@wur.nl (B.K.); henry.vandenbrand@wur.nl (H.v.d.B.); 2 Cargill Animal Nutrition Innovation Center, Cargill B.V., Veilingweg 23, 5334 LD Velddriel, The Netherlands

**Keywords:** stillbirth, pre-weaning mortality, nitrate, sow, farrowing, placenta

## Abstract

**Simple Summary:**

Over recent decades, the number of piglets and, therefore, the number of stillborn piglets per litter have been increasing. Blood and oxygen supply are crucial for piglets to survive the birth process. Blood flow might be increased through vasodilation by dietary nitrate supplementation, which is known in sports nutrition to increase endurance. The current study evaluated the effects of nitrate supplementation to sows on the incidence of stillbirth at a commercial farm. In total, 120 sows received either a control diet or a diet containing 0.1% of calcium nitrate from approximately 5 days until 4 days after farrowing. The number of piglets born alive, stillborn, or that died from birth to weaning were recorded. Piglets were weighed at birth, after cross-fostering, 24 h after cross-fostering, at 3 days of age, and at weaning. Placentas were collected after expulsion and were visually scored on redness. No effect of nitrate supplementation to the sow was found on placental redness, piglet weights, and growth or incidence of death after being born. Dietary nitrate supplementation decreased the stillbirth percentage from 9.9 to 7.4%, making it a potential approach to decrease stillbirth.

**Abstract:**

The objective of the current experiment was to investigate whether or not maternal dietary nitrate supplementation, a nitric oxide (NO) precursor, could reduce piglet losses under commercial circumstances. In the current experiment, 120 hyper prolific gilts and sows (Landrace x Yorkshire: Danbred) on a commercial farm in Denmark received either a control lactation diet or a lactation diet containing 0.1% of calcium nitrate (containing 63.1% of nitrate) from approximately 5 days pre-farrowing until day 4 of lactation. The number of piglets born total, alive, and stillborn, as well as birth weights, weights after cross-fostering (approximately 1 day of age), 24 h after cross-fostering, day 3 of age, and at weaning was recorded. Placentas of sows were collected after expulsion and scored on redness. No effect of nitrate supplementation was found on piglet weight, piglet growth, placental redness score, and pre-weaning mortality during lactation. Maternal dietary nitrate supplementation decreased stillbirth percentage with 2.5% (9.9 vs. 7.4%; *p* = 0.05). It can be concluded that maternal dietary nitrate supplementation shows the potential to decrease the incidence of stillbirth in hyper prolific sows.

## 1. Introduction

Stillborn piglets are a great loss and represent a welfare and societal issue for the pig industry [1]. In hyper prolific herds, such as in Denmark, the stillbirth percentage increased to an average of 10.2% in 2019 [2]. Stillbirths are typically associated with intra-uterine asphyxia or dystocia [3], in which the placenta and umbilical cord play a crucial role. Fetal asphyxia during birth can partially be explained by: (1) compression of the umbilical cord and placenta due to successive uterine contractions or when fetuses enter the pelvis [4], (2) loss of umbilical cord functionality (e.g., breaking, knots, wrapping around limps, stretching, etc.) [5,6], or (3) premature detachment of the placenta. Additionally, stillbirth is related to farrowing duration [7,8,9]. For example, Langendijk et al. [10] showed an increase in stillborn percentage from 2.7 to 10.7% and 27.3% when the farrowing duration increased from less than 2 h to 4–6 h and over 8 h, respectively.

Recently, the effects of maternal dietary interventions have been studied on farrowing duration and the risk of stillbirth [11,12,13,14,15,16]. Human sports supplements have been shown to induce vasodilatation, which increases blood flow and consequently oxygen flow in the body [17,18,19]. Consequently, performance can be enhanced by increasing stamina. It can be hypothesized that comparable effects can be obtained in sows around farrowing. By ensuring a larger blood flow and, consequently, oxygen and nutrient flow in the placenta and the umbilical cord during farrowing, the risk for asphyxiation and stillbirth can be reduced.

A potential candidate nutrient that might affect blood supply to the target tissue is nitrate [12,13]. Dietary nitrate has been shown to improve endurance exercise performance in human athletes [20,21,22]. Nitrate (NO_3_) in itself is inert, but after conversion to nitrite (NO_2_), mainly facilitated by bacteria in the mouth [23], it is further reduced to nitric oxide (NO, by denitrifying anaerobic bacteria or periodontal acidity [19,24]), which is a vasoactive component. The NO_3_-NO_2_-NO pathway is suggested to be very important in the regulation of blood flow [25], as shown by Larsen et al. [26]. They found a reduction in blood pressure in healthy volunteers when nitrate was supplemented in the diet for three days. In pigs, maternal dietary nitrate supplementation has, to our knowledge, only been studied by van den Bosch et al. [12,13] in a dose–response study, feeding up to 0.24% CaNO_3_ from 7 days pre-farrowing to 4 days post-farrowing. Piglet vitality, placental size, and piglet birth weight linearly increased with the dose of maternal nitrate supplementation [12,13].

It can be hypothesized that maternal nitrate supplementation could lead to an increased stamina of the sow and an adequate in utero blood flow during farrowing, due to the vasoactive properties of NO. Either one or both of these modes of action might reduce the duration of farrowing and decrease the incidence of stillbirth. The aim of this study was to evaluate the effects of maternal dietary nitrate supplementation around farrowing on the incidence of stillbirth, piglet performance, and pre-weaning mortality on a commercial farm with hyper prolific sows with a high incidence of stillbirth.

## 2. Materials and Methods

All experimental procedures were approved by the institutional animal use and care committee of Wageningen University and Research (Wageningen, The Netherlands).

### 2.1. Animals and Diets

The experiment was performed at a commercial farm in Holstebro, Denmark in 2015. In three consecutive batches, 134 hyper prolific crossbred sows (Landrace x Yorkshire: Danbred) were allocated based on parity (range 1 to 9) to one of two treatments containing 0.0% (control) or 0.1% of calcium nitrate (5Ca(NO_3_)_2·_NH_4_NO_3_.10H_2_O; containing 63.1% of nitrate; commercial name Bolifor CNF (Yara Phosphates Oy, Helsingborg, Sweden)) in the final diet. Two concentrates (10% of the final diets) for the control and calcium nitrate group were produced by Cargill Animal Nutrition (Rotterdam, The Netherlands). Calcium levels in the two concentrates were kept constant by exchanging limestone and calcium nitrate. Concentrate compositions are shown in Table 1. On the farm, these concentrates were mixed with other raw materials, as shown in Table 2, to obtain a final diet fed in a dry mash form. Consequently, sows in the treatment group received a maximum amount of nitrate of 32 mg/kg BW per day, which is considerably lower than the no observed adverse effect level (NOAEL) of 410 mg nitrate/kg BW per day, as indicated by the EFSA [27].

Experimental diets were fed twice a day (7.00 h and 15.00 h) from day 112 of gestation until 4 days after farrowing (based on the individual farrowing date of the sow). Diets were fed restrictedly at 3.8 kg/sow/d on day 112, 2.9 kg/sow/d on days 113 and 114, and 2.4 kg/sow/d on day 115 until the day of farrowing. After farrowing, diets were provided at 3.1, 3.7, 4.0, and 4.7 kg/sow/d at days 1, 2, 3, and 4 after farrowing, respectively. Starting at day 5 of lactation to weaning (day 23.6 ± 2.1), a commercially available lactation diet (15.2% CP, 9.3 MJ NE/kg) was provided 3 times a day (7.00 h, 12.00 h, and 15.00 h) to all sows in a liquid form. Sows had ad libitum access to drinking water. Piglets received potato starch until day 10 of age via floor feeding. From 10 days of age until weaning, a commercially available pre-starter (17.5% CP, 11.9 MJ NE/kg) was provided in a feeding bowl once per day.

### 2.2. Animal Housing and Management

Approximately 10 days before the expected farrowing date, pregnant sows were moved to individual farrowing pens with farrowing crates in 1 out of 5 farrowing rooms. Room 1 had 36 pens (used in round 1), room 2 had 24 pens (used in round 1), room 3 had 36 pens (used in round 2), room 4 had 34 pens (used in rounds 2 and 3), and room 5 also had 34 pens (used in round 3). All rooms had the same type of pens, farrowing crates, and flooring. All rooms had unblinded windows with natural light coming in. Pens contained concrete flooring with steel slats under the farrowing crate, located over a manure pit. Each pen contained a piglet nest with heated flooring, a heating lamp set at 30 °C, and saw dust. Piglets had ad libitum access to drinking water. Cross-fostering, all performed by the same person, was allowed within treatment groups until 3 days of age. The number and body weight of piglets that were cross-fostered and the date and time of cross-fostering were recorded. Litters were standardized to 14–15 piglets per sow, based on her mothering ability and number of functional teats. Leftover piglets were placed at foster sows, which were one week in lactation (sows not in experiment). To prevent errors in feeding or cross-fostering, treatments were allocated to the left or right side of the central corridor in each farrowing room. Per farrowing room, the allocation of treatment per side was performed randomly. Sows were allocated to treatments based on parity.

### 2.3. Measurements

Sow P2 backfat thickness (on the last rib, 6 cm down the dorsal middle line) was measured by the same person when sows were, on average, at day 112 of gestation and at weaning. Farrowing induction, medicine administration around or during farrowing, and use of birth assistance were recorded. If farrowing was completed at 6.00 a.m., the gestation length, total number of piglets born (TB), total number of piglets born alive (TBA), and total number of stillborn piglets (TSB, visually determined) were recorded. When sows finished farrowing during the time staff was present (between 5.30 a.m. and 4.00 p.m.), weighing took place on that day. Mummified and degenerated piglets were excluded from the total number born. Piglet weights were determined within 24 h after birth (daily at 6.00 a.m., before cross-fostering took place), after cross-fostering (between 7.30 a.m. and 9.00 a.m.), 24 h later, at 3 days of age, and at weaning. Time of weighing was recorded.

Number of dead piglets, reason for mortality (e.g., crushing, weak, starvation, diarrhea, and unknown), and weight of dead piglets were registered on a daily basis.

### 2.4. Placenta Analysis

Placentas of sows were collected during and after farrowing and stored at −20 °C. After thawing, each placenta was cut open over the whole length on the lateral side, using the umbilical cord as a reference. Open placentas were spread out on a white triplex board with the umbilical cord facing upward. Individual placentas were photographed in a room with standardized conditions (no natural light), using a Nikon D80 camera with a Nikon DX SWM ED IF aspherical 67 lens with fixed settings on height, zoom, color saturation, and ISO sensitivity settings. The color of the placenta was scored, using a scoring system from 0 to 4 adapted from Baxter et al. [28]. Scores were:

- 0 = No score possible or placenta was brown, because of deteriorating tissue.
- 1 = Placenta color was pale pink.
- 2 = Placenta color was light red or bright pink.
- 3 = Placenta color was bright red.
- 4 = Placenta color was deep red.

Placentas with a color score of 0 were removed from the analysis (19 placentas in total).

### 2.5. Statistical Analysis

One sow was aborted after being allocated in the current experiment. Data from sows that received birth assistance (*n* = 8), refused to eat (*n* = 2), farrowed too early (one day on feed) (*n* = 1), or had 8 or more stillborn piglets (*n* = 2, one in each treatment group) were removed from the dataset. The final dataset contained 120 sows. Parity was classified as class 1: parity 1; class 2: parity 2, 3, and 4; class 3: parity > 4. All variables were checked for normality on both means and residuals before analysis. TSB (ordinal data) was found to be nonnormally distributed even after data transformation and was expressed as a percentage of TB. Placental color scores were analyzed as ordinal data. Variables were analyzed with mixed models, using the PROC GLIMMIX procedure in SAS (version 9.3, 2011; SAS Institute Inc., Cary, NC, USA) according to the following statistical model:

Y_ijkl_ = *µ* + α_i_ + b_j_ + c_k_ + ε_ijkl_

where: Y_ijkl_ = dependent variable, *μ* = overall mean, α_i_ = fixed treatment effect (i = 0.0 or 0.1% of calcium nitrate), b_j_ = random effect of farrowing room (j = 1, 2,…, 5), c_k_ = random parity class effect (k = 1, 2 or 3), and ε_ijkl_ = residual error term. As the effects of batch and room were confounded, only room was added as a random effect to the model. For gestation length, stillborn rate, birth weights, and pre-weaning mortality rate, the random effect of days on the experimental diet before farrowing (l = 2, 3,…, 10) was added to the model. Backfat measurements at weaning were corrected for the number of days between measurements (covariable). Piglet birth weights were corrected for litter size (covariable) and piglet weaning weights were corrected for weaning age (covariable) and number of piglets weaned (covariable). Sow with litter was considered as the experimental unit. For analysis of placenta scores, the effect of sow (placenta) was added to the model as a random factor.

Preliminary analyses demonstrated a lack of effects of two potential interactions: (1) between litter size (TB) and treatment (control vs. calcium nitrate) and (2) between days on feed before farrowing and treatment. Consequently, results will be expressed per main effect of treatment.

Data are expressed as LSMeans and SEM, unless reported otherwise. Differences were assumed to be significant if *p* ≤ 0.05 and *p* > 0.05, but *p* < 0.10 was considered a trend.

## 3. Results

Average gestation length was 117.6 ± 1.2 days (taking the first day of insemination as day 1 of gestation), which led to sows being 5.2 ± 1.3 days on the experimental diets before the moment of farrowing. Mean TB was 18.2 ± 3.5 piglets per litter, with 16.6 ± 3.2 live born and 1.6 ± 1.5 (8.8%) stillborn (all mean ± SD). Piglets were weaned at 23.6 ± 2.1 days of age.

### 3.1. Sow Performance

A significantly longer gestation length was found for sows receiving the dietary nitrate (+0.4 days, *p* = 0.05, Table 3). This resulted in sows receiving the experimental diet significantly longer (+0.5 days, *p* = 0.03) than sows in the control group. No difference was found between treatments in backfat thickness of sows at approximately day 112 of gestation, at weaning and backfat loss during lactation (Table 3).

### 3.2. Piglet Weights and Average Daily Gain (ADG)

No effect of maternal dietary nitrate supplementation was found on the birth weight of live or stillborn piglets. In addition, no effect of maternal dietary nitrate supplementation was found on piglet ADG between cross-fostering and 24 h after cross-fostering, as well as between 24 h after cross-fostering and 3 days of age. Lastly, no effect of maternal dietary nitrate supplementation was found on weaning weight.

### 3.3. Piglet Survival

**Figure 1:**
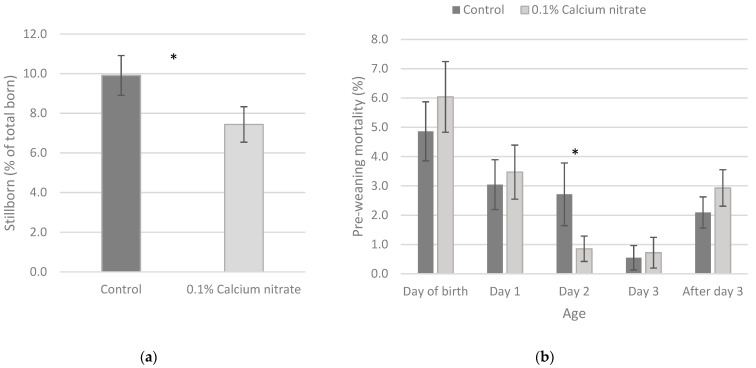
Stillborn percentage (**a**) and pre-weaning mortality percentage during the first three days of life (**b**) for piglets born out of sows receiving the control diet or the 0.1% calcium nitrate diet. * Indicates a significant difference (*p* < 0.05).

A significantly lower percentage of stillborn piglets was found when sows received 0.1% of calcium nitrate from approximately 5 days before farrowing onward compared to the control treatment (7.4 vs. 9.9%, respectively, *p* = 0.05, Figure 1a). Mortality was significantly lower on day 2 post-farrowing for piglets of sows receiving the dietary nitrate compared to the control (0.9% vs. 2.7%, respectively, *p* < 0.01, Figure 1b). However, on the day of birth, day 1, and day 3 of age, piglet mortality was non significantly (*p* > 0.05) higher for litters of which sows received the calcium nitrate compared to the control treatment. This resulted in a lack of effect of nitrate addition on the total pre-weaning mortality compared to the control treatment (15.3% vs. 14.3% for control and calcium nitrate, respectively, *p* = 0.55).

### 3.4. Placental Color Score

No effect of maternal dietary nitrate supplementation was found on placental color score, as shown in Table 4.

## 4. Discussion

Dietary nitrate supplementation to sows from approximately 5 days before farrowing until 4 days after farrowing resulted in a small but significantly longer gestation length, a lower stillbirth percentage, and a lower pre-weaning mortality rate in piglets at 2 days of age. Overall pre-weaning mortality was not affected by treatment. Placental redness score was not affected when dietary nitrate was supplemented to the maternal diet.

Average litter size and stillborn percentage were high in the current study (18.2 and 1.6, respectively) but comparable to the average production levels in Denmark in 2015 (e.g., 17.6 total born and 1.6 stillborn) [2]. The nitrate dosage in the current study corresponded with the 0.06% dosage, used in previous studies of Van den Bosch et al. [12,13]. In these studies, no significant effect of maternal nitrate supplementation was found on the incidence of stillbirth, which is in contrast to results found in the current study. It might be that the higher incidence of stillbirth in the current study (8.8%) provided more room for improvement compared to the study of Van den Bosch et al. [12] (5.8%). Sows in the current experiment were two days shorter on the feed before farrowing compared to the study of Van den Bosch et al. [12,13]. Based on studies in humans, it is hypothesized that the effects of nitrate supplementation quickly follow after ingestion. In humans, a rapid increase in plasma nitrate levels within the first 30 min after ingestion was found, with a peak occurring around 1.5–2 h after ingestion. Plasma nitrate levels stayed elevated for 6 to >11 h after ingestion [29,30,31] and may increase after each meal (resulting in increased saliva production, which contains nitrate), because nitrate is not only ingested when supplemented to the diet, but previously supplied nitrate is taken up by the salivary glands as well. It was estimated that about 25% of all plasma nitrate is taken up by the salivary glands and continuously secreted in saliva, where it is reduced to nitrite by commensal bacteria in the mouth and is then swallowed [25,29]. In human blood, nitrate has a half-life of 5 to 8 h [19,32] and nitrite (NO_2_^−^) has a half-life of 1 to 5 min [19]. NO is a highly reactive free radical with a half-life of only a few seconds. NO (or one of the reaction products) is quickly oxidized to arrange higher nitrogen oxide concentrations, such as nitrate and nitrite [33]. On commercial farms, most sows will receive either 2 or 3 meals per day or are fed ad libitum. Supplementation of a source of nitrate to the lactation diet of sows will most likely cause a sufficient increase in nitrate levels and therefore continuously synthesize nitrite and NO at the moment of farrowing to ensure the potential effects on endurance and vasodilatation, which may result in a reduction in stillbirth and an increase in piglet vitality. Consequently, it can be hypothesized that, although nitrate was supplemented shortly before the onset of farrowing, the timeframe of supplementation is sufficient to see effects.

Another major source of NO production is via the endogenous L-Arginine-NO synthase pathway, in which L-Arginine is oxidized via the nitric oxide synthase (NOS) family [34]. Only a few studies investigated the effects of maternal dietary arginine supplementation until right before the moment of farrowing. Che et al. [35] evaluated the supplementation of 1% of L-Arginine to sows from day 30 of gestation to day 114 of gestation and showed a significantly lower number of stillborn piglets (−0.6 pigs, *p* < 0.05) compared to the control. Mateo et al. [36] supplemented 1.0% of L-arginine to gilts from day 30 to 114 of gestation (TB was 11.6 piglets/gilt) and showed a significantly lower stillbirth (−1.2 pigs, *p* < 0.05). Gao et al. [37] found no significant difference in the absolute number of stillborn piglets (1.21 vs. 1.42, for the control and L-Arginine treatment, respectively), but this lack of effect might be related to the difference in total born piglets (12.46 vs. 13.77, for the control and L-Arginine treatment, respectively). Che et al. [35] hypothesized that the reduction in stillbirth can be due to increased utero-placental blood flow and maternal nutrient transfer, which supports a more efficient uterine capacity for fetal growth and development. When this physiological mechanism indeed occurs, it can be speculated that NO production via the maternal diet is particularly of interest in larger litters, because of the, on average, smaller placenta [28]. A one-to-one comparison between studies, using maternal L-arginine supplementation or maternal nitrate supplementation as a NO precursor, is not possible, as it is unknown how much NO is produced in the body by both supplements.

Although research on crosslinks between the NO_3_-NO_2_-NO pathway and the L-Arginine NO synthase pathway is limited, there are some indications that cross-talk exists between the two pathways in vascular NO homeostasis [38]. Lundberg et al. [39] suggested that NO generation from nitrite could be a back-up system for situations in which conditions for inducible NOS (iNOS) production is unfavorable (low oxygenation and acidification). Long-term (8–10 weeks) nitrate supplementation in rats showed a reversible dose-dependent reduction in phosphorylated endothelial NOS (eNOS) in the aorta and a lower eNOS-dependent vascular response in vessels from nitrate-treated mice [38], suggesting that, indeed, NOS activity is lower when nitrate is supplemented. Carlstorm et al. [38] suggested that mainly individuals (e.g., elderly) with a compromised eNOS activity might show an increased response to nitrate supplementation [38]. As, in the current study, dietary nitrate was only supplemented for a total of, on average, 9 days, a reduced eNOS activity is not expected to occur.

Gestation length was significantly longer when sows received dietary nitrate supplementation (+0.4 days, *p* < 0.05). It is difficult to say whether or not this is truly caused by treatment or a result of how gestation length was registered. Day of farrowing was noted when employees were present. Sows that farrowed in the evening when employees left were registered to have farrowed on the day after. In addition, a nonsignificant difference in litter size was observed between the control and nitrate supplementation treatment (18.7 vs. 17.9 piglets, respectively, *p* = 0.25). A negative correlation exists between litter size and gestation length [40,41], which is likely caused by an earlier occurrence of fetal stress caused by space limitation due to fetal mass in the uterus, which induces the onset of parturition [42].

An absolute 2.5% reduction in stillbirth percentage (*p* = 0.05) was found when nitrate was supplemented to sows compared to the control treatment. The reduction in stillbirth percentage might be a result of a shorter duration of farrowing caused by an increased stamina. Farrowing duration is directly linked to the incidence of stillbirth [7,8,9]. However, farrowing duration could not be registered in this experiment. Van den Bosch et al. [13] did not find an effect of maternal dietary nitrate supplementation on the duration of farrowing in a dose–response study, which was hypothesized to be due to the short duration of farrowing observed in that study and, therefore, little room for improvement. As a significant effect of maternal nitrate supplementation was found on gestation length, it can be hypothesized that the lower stillbirth percentage was caused by this increase in gestation length. The literature mainly describes an effect of gestation length on stillbirth when the gestation length is short (<114 days) [40,43,44]. Rydhmer et al. [44] showed a linear decrease in the number of stillborn piglets from day 111 until 120 of gestation with a nonsignificant difference between day 117 and 118 of gestation. It therefore seems unlikely that an increased gestation length of 0.4 days is the driver for a 2.5% reduction in stillbirth percentage. In addition, the number of days on feed, which is confounded with gestation length, was added to the statistical models to correct for potential effects. No effect of treatment on the incidence of pre-weaning mortality during the whole lactation period was found. Van den Bosch et al. [12] found a trend for a quadratic effect of nitrate dosage on pre-weaning mortality percentage, with the lowest percentage seen at approximately 0.09–0.12% of nitrate. NO is capable of relaxing the vascular endothelium, causing vasodilatation [17,45], which may have led to a larger blood flow and, consequently, oxygen flow to the fetuses in utero. This larger oxygen flow might have reduced the level of asphyxiation in piglets, causing piglets to be born more vital and therefore reducing the risk for mortality. Van den Bosch et al. [13] showed a trend (*p* = 0.10) for an increased partial oxygen pressure (*p*O_2_) in umbilical cord blood of newly born piglets when an increasing dose of maternal dietary nitrate was fed. In addition, piglet vitality score increased linearly with the dosage of maternal nitrate supplementation, which might have been caused by the increased placenta size observed [13] and/or the increased birth weight of piglets [12]. Why no clear effect on pre-weaning mortality was found in the current experiment might be related to the use of cross-fostering. Cross-fostering is a common technique used to match the litter size of a sow to her mothering ability [46]. Cross-fostering piglets to another sow has an impact on the environment of the fostered piglet, as well as on the litter this piglet is fostered onto.

No effect of maternal nitrate supplementation was found on placental redness score, which is in line with the study of Van den Bosch et al. [13]. As mentioned before, NO is an endothelial derived relaxing factor, which regulates blood flow across tissues (including the uterus and placenta) and, consequently, the nutrient and oxygen flow from the mother to fetuses [17,45]. NO also enhances placental vascular growth by placental angiogenesis. Uterine and umbilical cord blood flow increases exponentially throughout gestation to keep up with increasing fetal growth [47], which means the uterine and placental vascular wall keep remodeling to provide this essential blood flow [48]. It was therefore expected that a higher placental redness score could be observed in placentas of sows receiving maternal dietary nitrate supplementation. It might be that due to the short time of nitrate supplementation (e.g., approximately 5 days pre-farrowing), the time frame to adapt the placental vascular system was too short and only vasodilation might occur. Widening of the blood vessels could potentially not be visible anymore after placenta expulsion. In addition, placentas were collected after expulsion deprived from the maternal circulation for some time. The dead placenta tissue may not have given us the required information on vasodilatation. Vascularization of the placentas, due to maternal nitrate supplementation, has not been studied in the current experiment and could therefore be a topic for future research.

## 5. Conclusions

Maternal dietary nitrate supplementation from approximately 5 days before farrowing reduced the incidence of stillbirth with 2.5% in hyper prolific sows under commercial circumstances. No effect of maternal dietary nitrate supplementation was found on piglet (birth) weights, growth, placenta redness score, or incidence of pre-weaning mortality during lactation. It can be concluded that maternal dietary nitrate supplementation shows the potential to decrease the incidence of stillbirth in hyper prolific sows.

## 6. Patents

A related patent application (PCT/US2015/064293) was filed on 7 December 2015, and accepted as WO/2016/090366 on 9 April 2021.

## Figures and Tables

**Table 1:** Composition of the experimental concentrates (10% inclusion in the final diet), as formulated.

Ingredients (%)	Control	0.1% CaNO_3_	Nutrient Levels	Control	0.1% CaNO_3_
Provisoy	50.000	50.000	Dry Matter (%)	94.64	94.50
Chicory Pulp	10.000	10.000	NE (MJ/kg)	7.011	7.095
Calcium Carbonate	7.351	6.856	Crude protein (%)	28.50	29.95
Monocalcium Phosphate	6.888	6.884	AID Lys (%)	1.933	1.933
Wheat	5.797	6.602	AID Met + Cys (%)	1.141	1.146
Salt	3.757	3.454	Calcium (%)	4.61	4.61
Potassium Chloride	3.358	3.449	Phosphorus (%)	1.90	1.90
Choline Chloride 60%	-	2.500	Sodium (%)	2.20	1.68
Choline Chloride 70%	2.143	-	Potassium (%)	2.89	2.89
Sodium Bicarbonate	2.479	1.056	Magnesium (%)	1.26	1.26
Bolifor CNF ^1^	-	1.000			
Soya Oil	2.000	2.000			
L-Lysine HCL	0.439	0.417			
DL-Methionine	0.396	0.391			
Commercial Premix ^2^	5.393	5.393			

^1^ Bolifor CNF, available from Yara Phosphates Oy, consists of calcium nitrate (5Ca(NO_3_)_2·_NH_4_NO_3_.10H_2_O); containing 63.1% of nitrate. ^2^ Commercial sow premix from Cargill Animal Nutrition. – Means not added.

**Table 2:** Calculated composition of the experimental diets as mixed on farm (as fed).

Ingredients (%)	Control	0.1% CaNO_3_
Barley	48.50	48.50
Wheat	25.00	25.00
Beet pulp, sugar 5.9%	8.80	8.80
Soybean meal, dehulled	7.00	7.00
Soybean oil	0.70	0.70
Concentrate Control	10.00	-
Concentrate CaNO_3_ ^1^	-	10.00

^1^ Bolifor CNF, available from Yara Phosphates Oy, consists of calcium nitrate (5Ca(NO_3_)_2_·NH_4_NO_3_.10H_2_O); containing 63.1% of nitrate. – Means not added.

**Table 3:** Effects of calcium nitrate (0.1% Bolifor CNF) in the maternal diet of sows, fed from approximately 5 days pre-partum until 4 days post-partum on sow backfat thickness, reproductive performance, and piglet weight.

Variable	Control	0.1% Calcium Nitrate ^1^	*p*-Value
*N (number of sows/litters)*	63	57	-
Parity before farrowing	3.3 ± 0.2	3.5 ± 0.3	0.70
Gestation length (days)	117.0 ^b^ ± 0.5	117.4 ^a^ ± 0.5	0.05
Number of days on feed before farrowing (days)	4.5 ^b^ ± 0.5	5.0 ^a^ ± 0.5	0.03
*Sow backfat thickness*			
At approximately day 112 of gestation (mm)	15.0 ± 0.8	15.5 ± 0.8	0.37
At weaning (mm) ^2^	12.6 ± 0.6	12.7 ± 0.6	0.88
Backfat loss during lactation (mm) ^2^	2.8 ± 0.5	3.2 ± 0.5	0.14
*Reproductive performance*			
Total born	18.7 ± 0.5	17.9 ± 0.5	0.25
Number of piglets after cross-fostering	14.7 ± 0.6	15.0 ± 0.6	0.59
Number of piglets weaned	12.7 ± 0.2	12.6 ± 0.2	0.38
*Piglet weights and ADG*			
Birth weight live-born piglets (kg) ^3^	1.34 ± 0.09	1.33 ± 0.09	0.76
Birth weight stillborn piglets (kg) ^3^	1.08 ± 0.07	1.08 ± 0.07	0.97
Average weight after cross-fostering (kg)	1.23 ± 0.09	1.25 ± 0.10	0.65
Average weaning weight (kg) ^4^	6.06 ± 0.62	6.21 ± 0.63	0.48
ADG cross-fostering until 24 h after cross-fostering (g/piglet/day)	87 ± 13	92 ± 13	0.65
ADG from 24 h after cross-fostering until 3 days of age (g/piglet/day)	119 ± 19	121 ± 19	0.79

^1^ Calcium nitrate ((5Ca(NO_3_)_2_.NH_4_NO_3_.10H_2_O); containing 63.1% of nitrate; commercial name Bolifor CNF, available from Yara Phosphates Oy). ^2^ Corrected for number of days between BF measurements. ^3^ Corrected for total number born. ^4^ Corrected for total number weaned per litter and weaning age. ^a, b^ Different superscripts indicate a significant difference (*p* ≤ 0.05) between treatments.

**Table 4:** Frequencies of placental color score for the control and 0.1% of calcium nitrate group.

Score ^1^	Control		0.1% Calcium Nitrate		Pooled SEM	*p*-Value
	n ^2^	%	n	%		
					3.2	0.19
1	27	7.8	41	12.1		
2	145	41.9	135	39.8		
3	146	42.2	144	42.5		
4	28	8.1	19	5.6		
Total	346	100.0	339	100.0		

^1^ Color of the placenta was scored on a 0 to 4 scale adapted from Baxter et al. [28]. 1 = Placental color was pale pink. 2 = Placental color was light red or bright pink. 3 = Placental color was bright red. 4 = Placental color was deep red. ^2^ Represents the number of placentas, but data analyzed on sow basis.

## Data Availability

The data presented in this study are available on request from the corresponding author.
